# Close relatives in population samples: Evaluation of the consequences for genetic stock identification

**DOI:** 10.1111/1755-0998.13131

**Published:** 2020-01-27

**Authors:** Johan Östergren, Stefan Palm, John Gilbey, Johan Dannewitz

**Affiliations:** ^1^ Department of Aquatic Resources Institute of Freshwater Research Swedish University of Agricultural Sciences Drottningholm Sweden; ^2^ Marine Scotland Science Freshwater Fisheries Laboratory Pitlochry UK

**Keywords:** colony, family sampling, genetic differentiation, individual assignment, mixed stock analysis, *Salmo salar*

## Abstract

Determining the origin of individuals in mixed population samples is key in many ecological, conservation and management contexts. Genetic data can be analyzed using genetic stock identification (GSI), where the origin of single individuals is determined using Individual Assignment (IA) and population proportions are estimated with Mixed Stock Analysis (MSA). In such analyses, allele frequencies in a reference baseline are required. Unknown individuals or mixture proportions are assigned to source populations based on the likelihood that their multilocus genotypes occur in a particular baseline sample. Representative sampling of populations included in a baseline is important when designing and performing GSI. Here, we investigate the effects of family sampling on GSI, using both simulated and empirical genotypes for Atlantic salmon (*Salmo salar*). We show that nonrepresentative sampling leading to inclusion of close relatives in a reference baseline may introduce bias in estimated proportions of contributing populations in a mixed sample, and increases the amount of incorrectly assigned individual fish. Simulated data further show that the induced bias increases with increasing family structure, but that it can be partly mitigated by increased baseline population sample sizes. Results from standard accuracy tests of GSI (using only a reference baseline and/or self‐assignment) gave a false and elevated indication of the baseline power and accuracy to identify stock proportions and individuals. These findings suggest that family structure in baseline population samples should be quantified and its consequences evaluated, before carrying out GSI.

## 1 INTRODUCTION

Determining the population origin of individuals is fundamental in many ecological, evolutionary, conservation, and management contexts (e.g., Allendorf & Luikart, 2007). For example, to allow efficient management and conservation, it is critical to study population specific harvest rates of exploited wild animals, in particular endangered ones. In fisheries science, identification of individuals or population (stock) proportions is particularly important since exploitation often take place in mixed‐stock fisheries (Carvalho & Hauser, 1994; Hilborn, Quinn, Schindler, & Rogers, 2003). Knowledge on stock specific harvest rates or catch composition can be used to preserve intraspecific genetic diversity, as it allows managers to selectively harvest stocks according to their relative abundance and productivity.

Assigning stocks or individuals to putative sources of origin can be done using various methods or techniques (Cadrin, Friedland, & Waldman, 2005), such as tagging data (e.g., Beacham et al., 2019; Brodziak, 1993), parasites (e.g. MacKenzie & Abaunza, 1998), age structure (e.g., Chasco, Hilborn, & Punt, 2007) and morphometric landmarks (Cadrin, 2000). However, over the past decades genetic methods have been increasingly used when assessing fish stock origin (see Hansen, Kechnington, & Nielsen, 2001; Manel, Gaggiotti, & Waples, 2005; Shaklee, Beacham, Seeb, & White, 1999; Whitlock et al., 2018 with references). Such analyses are collectively referred to as genetic stock identification (GSI) which includes techniques to estimate population proportions in a mixture (Mixed Stock Analysis, MSA) and the origin of single individuals (Individual Assignment, IA). The practice of using GSI for fisheries management purposes began in the 1980s (Pella & Milner, 1987) when genotypes at multiple loci (allozymes) were becoming commonly available. More recently an increasing number of studies analyzing stock mixtures in fisheries have been published (Beacham et al., 2018; Bradbury et al., 2016; Gilbey et al., 2017; Östergren, Nilsson, Lundqvist, Dannewitz, & Palm, 2016; Vaha, Erkinaro, Falkegard, Orell, & Niemela, 2017; Whitlock et al., 2018).

In MSA, genotypes in a mixed sample are compared to the expected genotype frequencies in a representative reference baseline containing potential originator populations or stocks, and the most likely population proportions (with surrounding uncertainty) are estimated statistically. Similarly, IA uses a genetic reference baseline to identify the most likely population origin of single individuals based on the likelihood of their multilocus genotype occurring in each reference sample. For MSA and IA both maximum likelihood and Bayesian approaches are available (Anderson, Waples, & Kalinowski, 2008; Cornuet, Piry, Luikart, Estoup, & Solignac, 1999; Pella & Masuda, 2001). The power and accuracy of GSI depends on several factors (e.g., Hansen et al., 2001) including degree of genetic differentiation between baseline populations, number and quality of markers, number of alleles (Beacham et al., 2005; Cornuet et al., 1999), temporal genetic stability, and the size of both the mixed fishery and reference baseline population samples (Beacham et al., 2006; Beacham, Mcintosh, & Wallace, 2011).

One aspect of particular importance is that the baseline adequately represents the allele frequencies in the source populations of interest. Sampling a species with high fecundity and/or when individuals occur in a nonrandom familial spatial distribution, requires a well‐planned sampling design to avoid over‐representation of close relatives or family members (e.g., full‐siblings). In the wild, such family sampling might occur when sampling newly hatched juveniles at a specific time period or in a limited space (e.g., a short stretch of a river) (Hansen, Nielsen, & Mensberg, 1997). Similarly, in fish hatchery environments, a limited number of adults are often used as broodstock, and offspring may be kept in tanks or trays which may hold just a few out of all families. In such situations, sampling design is crucial, as the risk of family sampling is obvious. In addition, survival rates among families can be highly variable both in wild and hatchery environments.

Recently, increased theoretical attention has been drawn to potential effects of close relatives (i.e., family structure) on common population genetics analyses, including estimates of allele frequencies, *F*‐statistics, expected heterozygosity (*H*_e_), effective and observed numbers of alleles, and tests for deviations from Hardy‐Weinberg (HWE) and linkage equilibrium (LE). Using computer simulated data, Wang (2018) showed that inclusion of excessive close relatives in samples upwardly biased estimates of *F*_ST_, reduced the value of *H*_e_ (given the same sample size with and without siblings), and induced Hardy‐Weinberg and linkage disequilibria. Waples and Anderson (2017) addressed problems that can arise when routinely removing putative siblings from samples before performing population genetic analyses, and showed that such purging can degrade precision of estimates of allele frequency and *F*_ST_ and bias estimates of effective population size (*N*_e_). They suggested that removal of siblings should be performed on a case‐by‐case basis, as it is difficult to make generalizations about specific situations.

The effects of close relatives on unsupervised Bayesian clustering algorithms using structure (Pritchard, Stephens, & Donnelly, 2000), which can be used without baseline samples to identify individuals or groups of populations in a mixed sample (e.g., Manel et al., 2005), has also been studied in detail (Anderson & Dunham, 2008; Rodriquez‐Ramilo & Wang, 2012). It was concluded that the clustering algorithm may overestimate the number of inferred clusters, when close relatives are present. The suggestion was therefore to identify and remove excessive full‐siblings before clustering analysis, since that may improve the ability of the algorithms to infer the correct number of population clusters.

Although the influence of family structure on various population genetic metrics has been investigated, there has to date been little focus on the influence of family structure on GSI (but see Banks, Rashbrook, Calavetta, Dean, & Hedgecock, 2000). Neither Wang (2018) nor Waples and Anderson (2017) specifically addressed genetic assignment tests in their theoretical evaluations based on simulated data, although the latter authors highlighted the need for empirical evaluations of effects of full‐siblings (and on removing them) on MSA and IA. We have only found one empirical study that explicitly analyzed the effect of coancestry between individuals on their assignment to a baseline (Guinand et al., 2006), but that study of lake trout (*Salvelinus namaycush*) in the Great Lakes did not investigate MSA or the effect of inclusion of close relatives in a baseline.

A common feature of software designed specifically for MSA and IA is the option to evaluate the reference baseline using a variety of simulations. Such analyses are commonly performed as a starting point in empirical studies to help to define the power of the reference baseline, and to allow for reliable estimates of stock proportions or assignment of individual fish. Self‐assignment tests remove each individual from the baseline and assign it back to the most likely population origin, while the 100% simulation test consists of simulating mixture genotypes from one population at a time, followed by estimates of their occurrence in the baseline populations. Potentially, both these standard accuracy tests may be affected by family structure in baseline samples.

**Figure 1:**
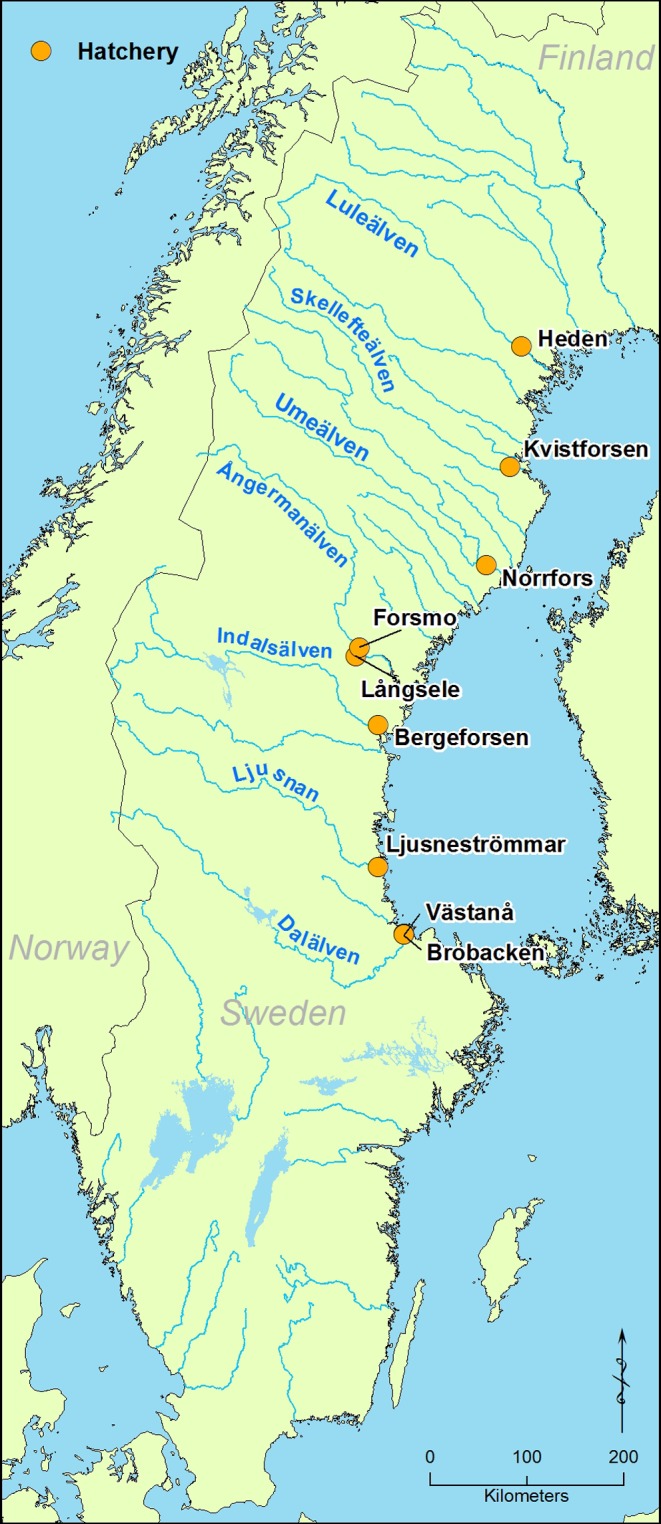
Map showing the geographic location of fish hatcheries where fish were sampled. River and hatchery names are also given in Table 1

In the Baltic Sea, fishing on Atlantic salmon (*Salmo salar*) is mainly undertaken in the southern Main basin and along the coasts of Sweden and Finland (Figure 1). In those areas, salmon from several wild and hatchery reared populations are exploited in mixed‐stock fisheries (Karlsson & Karlström, 1994; Siira, Erkinaro, Jounela, & Suuronen, 2009) and knowledge on stock‐specific harvest rates is therefore central for conservation and management (Östergren et al., 2015; Whitlock et al., 2018). In total, about five million hatchery reared salmon smolts (1 and 2‐year‐old juveniles) are released annually into the Baltic Sea as mitigation for reproduction losses due to hydroelectric power production (ICES, 2018). This amount is almost twice as high as the annual natural production during the last decade (approximately 2–3 million wild smolts per year) (ICES, 2018). Large‐scale tagging programmes of released reared salmon have been undertaken since the 1950s (Carlin, 1955), and previous studies on recaptured tags have investigated stock specific harvesting (e.g., ICES, 2018). In addition to the use of Carlin‐tags, which in the Baltic mainly gives information on reared stocks, there has been an increasing use of molecular techniques during the last 15 years to identify catch composition of both hatchery and wild stocks using genetic MSA. Such analysis on salmon have been performed on several occasions (Koljonen, 2006; Koljonen & McKinnell, 1996; Koljonen, Pella, & Masuda, 2005; Östergren et al., 2015, 2014; Whitlock et al., 2018), and is carried out on an annual basis within the work of ICES WGBAST (e.g., ICES, 2018).

To fill the knowledge gap on how close relatives in baseline population samples affects GSI, we investigated the effects of family structure in baseline population samples on the performance of GSI methods (MSA and IA). We approached these questions by analyzing empirical baselines from seven hatchery reared Atlantic salmon populations in the Baltic Sea combined with a complementary simulation exercise. Our key questions were as follows: (a) What are the effects of various degree of family structure in baseline population samples on GSI estimates (IA and MSA)? (b) Does the baseline population sample size influence the results at various degree of family structure? (c) How are commonly used tools for evaluation of baselines (e.g., self‐assignment and 100% simulations) affected by a varying degree of family structure in baselines? (d) What is the best way to mitigate the potential effects of family structure on GSI estimates?

## 2 MATERIALS AND METHODS

### 2.1 Study design

#### 2.1.1 Empirical study

**Figure 2:**
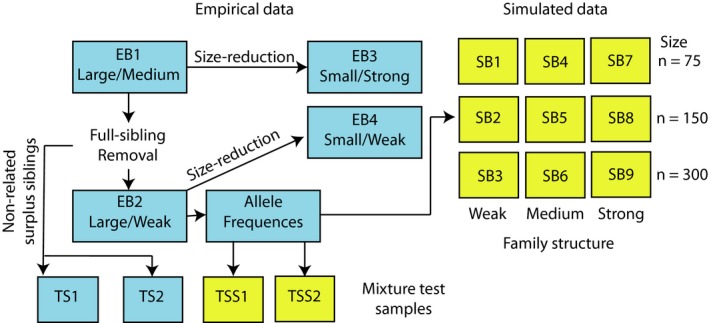
Flowchart illustrating the study design. Boxes represent baselines evaluated based on empirical (EB1–EB4, blue) and simulated data (SB1–SB9, yellow), and empirical (TS1 & TS2, blue) and simulated (TSS1 & TSS2, yellow) mixture test samples. EB1 includes all original baseline population samples. The sizes (small/*n* = 75, medium/*n* = 150, large/*n* = 300) and the level of family structure (weak, medium, strong) of baseline population samples is indicated. For EB1 (Large/Medium) and EB2 (Large/Weak) the baseline population samples size (large) are ranges for the seven populations included in the baselines (see Table 2). Empirical mixture test samples (TS1 & TS2) were created from nonrelated surplus siblings. Simulated baselines and mixture test samples (TSS1 & TSS2) were created with colony using allele frequencies in the baseline EB2 (Large/Weak), i.e., the large empirical baseline with one individual per full‐sibling family

The empirical data set, in total 1,870 fish, comprised of individuals (mainly juveniles) from seven hatchery reared stocks of salmon used for compensatory release in Swedish Baltic Sea rivers impacted by hydroelectricity schemes (Figure 1). In total, nine hatcheries were sampled, since two rivers had two hatcheries each (but use the same broodstock), and sampling took place at two occasions, in 2006 and 2013/2014 (Table 1). The study design is outlined below (Figure 2). In brief, the empirical data was first analyzed for the existence of family structure (i.e., full‐siblings) using the software colony (Wang, 2004; Wang & Santure, 2009) (see Appendix S1). We then pooled temporal baseline population samples from the same population, in accordance with recommendations by Waples (1990). Full details of all reference baselines can be found in Figures S1‐S5.

**Table 1:** Baseline population samples included in the study indicating sample year and sampled life stage

River (hatchery)	Baseline sample (*n*)			Sampled life stage
	2006	2013	2014	
Ångermanälven (Forsmo & Långsele)^a^	59		149	J + J
Dalälven (Brobacken & Västanå)^a^	116		128	J + J
Indalsälven (Bergeforsen)	119	246		J + A
Ljusnan (Ljusneströmmar)	119	214		J + A
Luleälven (Heden)	119		156	J + J
Skellefteälven (Kvistforsen)	119	82		J + J
Umeälven (Norrfors)	117		127	J + J

a Sampling took place at two hatcheries. J, juveniles; A, adults.

Following temporal pooling, four empirical baselines were defined (EB1–EB4; Figure 2). The first baseline (EB1) consisted of all sampled individuals and thus contained the original family structure, which mirrored how sampling had been performed in hatcheries. We defined this baseline to have a medium family structure (relative to additional baselines in this study). In the second baseline (EB2), defined to have a weak family structure, we excluded full‐siblings (FS) inferred by colony from all samples (i.e., we just kept one individual per inferred FS family). In the third and fourth baselines (EB3 & EB4) we reduced the maximum number of individuals per sample to 75 (from originally 201–365 per stock; Table 1). In baseline EB3, we created a strong family structure by keeping large family groups in all samples. Because there was different family group sizes in the different empirical baseline population samples, the EB3 baseline varied in terms of both number of individuals and number (and size) of families per sample (see Table 2 and Figure S3). In baseline EB4 we created a weak family structure of randomly selected nonrelated individuals from baseline EB2; hence, this baseline consisted of equal numbers (and sizes) of families (*n* = 75, Figure S4) with the exception of river population Skellefteälven (*n* = 58) where the number of families available was lower. For clarity reasons, we hereafter refer to the four empirical baselines using their codes and also their sizes/levels of family structure: EB1 (Large/Medium), EB2 (Large/Weak), EB3 (Small/Strong), and EB4 (Small/Weak) (see also Figure 2).

**Table 2:** Summary statistics for baselines used when testing GSI performance of pooled temporal samples before (EB1 [Large/Medium]) and after (EB2 [Large/Weak]) reduction of full‐siblings, and after sample size reduction (EB3 [Small/Strong] and EB4 [Small/Weak])

Baseline	Hatchery population	Sample size (*n*)	*N* Fam	Unbiased Hz (*H*_e_)		Ave. No Alleles	*F*_IS_	*p*‐value *F*_IS_
EB1 (Large/Medium)	Ångermanälven	208	79	0.7183	10.59		0.004	n.s.
EB2 (Large/Weak)		79	79	0.7179	10.24		0.002	n.s.
EB3 (Small/Strong)		75	24	0.7156	9.24		0.002	n.s.
EB4 (Small/Weak)		75	75	0.7180	10.18		0.007	n.s.
EB1 (Large/Medium)	Dalälven	244	97	0.7411	10.35		0.007	n.s.
EB2 (Large/Weak)		97	97	0.7411	10.12		0.012	n.s.
EB3 (Small/Strong)		75	11	0.7311	7.88		–0.009	n.s.
EB4 (Small/Weak)		75	75	0.7439	9.47		0.004	n.s.
EB1 (Large/Medium)	Indalsälven	365	144	0.7117	10.00		–0.007	n.s.
EB2 (Large/Weak)		144	144	0.7137	9.88		–0.015	n.s.
EB3 (Small/Strong)		75	13	0.6979	7.29		–0.008	n.s.
EB4 (Small/Weak)		75	75	0.7054	9.18		–0.02	n.s.
EB1 (Large/Medium)	Ljusnan	333	135	0.7295	10.88		–0.009	n.s.
EB2 (Large/Weak)		135	135	0.7286	10.71		–0.015	n.s.
EB3 (Small/Strong)		75	5	0.7027	6.35		–0.014	n.s.
EB4 (Small/Weak)		75	75	0.7277	9.71		0.003	n.s.
EB1 (Large/Medium)	Luleälven	275	131	0.7258	11.47		0.01	n.s.
EB2 (Large/Weak)		131	131	0.7209	11.24		–0.004	n.s.
EB3 (Small/Strong)		75	15	0.7243	8.88		0.001	n.s.
EB4 (Small/Weak)		75	75	0.7207	10.76		–0.006	n.s.
EB1 (Large/Medium)	Skellefteälven	201	58	0.7147	9.06		–0.015	*
EB2 (Large/Weak)		58	58	0.7251	8.59		0.015	n.s.
EB3 (Small/Strong)		58	5	0.6760	6.12		–0.111	***
EB4 (Small/Weak)		58	58	0.7251	8.59		0.015	n.s.
EB1 (Large/Medium)	Umeälven	244	87	0.6913	10.76		0.005	n.s.
EB2 (Large/Weak)		87	87	0.6962	10.06		–0.003	n.s.
EB3 (Small/Strong)		75	19	0.6913	8.06		0.018	n.s.
EB4 (Small/Weak)		75	75	0.6956	9.94		0.001	n.s.

Note

Estimates of unbiased heterozygosity, average number of alleles, *F*_IS_ with corresponding *p*‐values are shown (calculated based on 2,380 randomisations).

F_IS_ is shown with its level of significance (*** *p* < 0.001, ** *p* < 0.01, * *p* < 0.05, n.s. non‐significant).

In addition to the above four baselines, we constructed two empirical mixture test samples with known river origin for an evaluation of GSI performance. The two empirical mixture test samples (TS1 & TS2) consisted of non‐related individuals not used in the baseline EB3 (Small/Strong) or EB4 (Small/Weak) (i.e. surplus individuals purged when decreasing sample sizes) (Figure 2). TS1 consisted of equal number of individuals (*n* = 22) from four populations whereas TS2 consisted of unequal number of individuals (*n* = 4–30) from six populations (Figure 2, Table 3).

**Table 3:** Mixed‐fishery files used for testing GSI on empirical individuals (TS1 & TS2) and simulated genotypes of known origin using the baseline EB2 (Large/Weak) (TSS1 & TSS2). See text for details

	TS1 & TSS1		TS2 & TSS2	
	*n*	%	*n*	%
Ångermanälven	0	0	4	0.05
Dalälven	22	0.25	8	0.09
Indalsälven	22	0.25	30	0.34
Ljusnan	22	0.25	20	0.23
Luleälven	22	0.25	14	0.16
Skellefteälven	0	0	0	0
Umeälven	0	0	12	0.14

#### 2.1.2 Simulation study

In the simulation study, we defined nine baselines (SB1–SB9, Figure 2) simulated using colony (Appendix S1) based on the empirical allele frequencies in baseline EB2 (Large/Weak). The baseline population samples in the nine simulated baselines consisted of three levels of family structure (weak, medium and strong) combined with three sample sizes (75, 150 and 300 individuals per baseline population sample). The degree of family structure was defined as follows; weak – the same as in the EB2 (Large/Weak) baseline in the empirical study (i.e. non‐related individuals), medium – same average family structure as in baseline EB1 (Large/Medium), and strong – the strongest family structure in the empirical data, which was an equal family size of 15 individuals per baseline sample. The family structure in SB1–SB9 is graphically depicted in Figure S5.

Also in the simulation study we used two mixture test samples (TSS1 & TSS2, Figure 2). Those consisted of nonrelated individuals, simulated with colony using allele frequencies in the baseline EB2 (Large/Weak). Similar to the empirical study, TSS1 consisted of an equal number of individuals (*n* = 22) from four populations, whereas TSS2 consisted of unequal number of individuals (*n* = 4–30) from six populations (Figure 2, Table 3).

### 2.2 Genetic analyses

Tissue samples consisted of fin clips from hatcheries stored individually in labeled tubes with ethanol (95%). DNA was extracted followed by PCR and genotyping of 17 polymorphic microsatellite markers (on average c. 10 alleles/locus; Table 2). Baseline population samples from 2006 were genetically processed in Finland (Jarmo Koskiniemi, University of Helsinki), whereas baseline population samples from 2013/2014 (from broodstocks) were analysed in Sweden (SLU Aqua). To assure fully comparable genotypes scored at the two laboratories, a marker calibration (i.e., replicated allele length scoring of same individuals) was performed. Details on laboratory procedures, microsatellites screened and marker calibrations are provided as supportive information in Whitlock et al. (2018).

Part of the Swedish baseline population samples used in this study (<40%) were also used in the study by Whitlock et al. (2018). A test for repeatability and error rate at scoring of alleles at the SLU Aqua laboratory was performed on ethanol‐stored fin‐clips from two baseline population samples; approximately 10% (*n* = 30) of the individuals from Ljusnan and Skellefteälven sampled in 2013 were reanalysed de novo (from DNA extraction to scoring of alleles).

### 2.3 Statistical analysis

We used the maximum likelihood approach implemented in the computer software colony 2.0.4.4 (Wang, 2004; Wang & Santure, 2009) to identify full‐siblings in each of the empirical baseline population samples. colony was also used to simulate baseline and test sample data for the simulation study (see details in Appendix S1).

The program fstat (Goudet, 1995) version 2.9.3.2 was used to estimate expected heterozygosity (*H*_e_), *F*_IS_ and pairwise *F*_ST_ (Weir & Cockerham, 1984). The same program was used to conduct statistical tests for deviations from Hardy‐Weinberg equilibrium (2,380 randomisations) and genetic differentiation between pairs of samples (21,000 randomisations).

### 2.4 Evaluation of GSI performance

We used the program oncor (Kalinowski, Manlove, & Taper, 2007) for evaluating the GSI performance of each baseline, divided into two approaches: MSA and IA. All analyses with oncor were performed similarly for empirical and simulated data, as outlined below. Throughout, we applied the program default settings with 1,000 bootstraps.

We decided to use oncor for this study based on two main criteria; (a) It is/has been widely used in GSI studies and (b) is user‐friendly and has several built‐in simulation tests (e.g., 100% simulation and self‐assignment‐test) very commonly used in published GSI studies. Several other computer programs developed for GSI analysis exists, e.g., cbayes (Neaves, Wallace, Candy, & Beacham, 2005), geneclass2 (Piry et al., 2004), rubias (Moran & Anderson, 2019) and spam (Debevec et al., 2000), but a comparison of outcomes from different software was beyond the scope of this study. Furthermore, when evaluated in other studies, oncor has been shown to perform equal to several of those alternative software (Debevec et al., 2000; Griffiths et al., 2010; Ikediashi, Billington, & Stevens, 2012; Vaha et al., 2017). Therefore, we believe that our approach would have produced similar results independent of computer program used.

#### 2.4.1 Mixed Stock Analysis

First, we performed commonly used tests for power of the baselines to accurately estimate stock proportions, 100% simulations (Kalinowski et al., 2007), by simulating pure mixture samples from each baseline population (mixture sample size *n* = 200, number of simulations = 100, baseline sample size same as empirical baseline). As oncor uses allele frequencies in all baseline population samples (one at the time) to create mixture samples, effects from family structuring is passed on from empirical to simulated data. After MSA of the simulated 100% mixture files, we used the average results to evaluate accuracy. Second, we performed MSA on our predefined mixture test samples for the empirical (TS1 & TS2) and simulated (TSS1 & TSS2) data.

#### 2.4.2 Individual assignment

With oncor we initially performed a self‐assignment test with a leave‐one‐out (LOO) procedure to evaluate how accurate individual fish can be assigned to their population of origin. For self‐assignment, oncor assigns individuals with complete genotypes to putative sources of origin. Assignment accuracy for each baseline was evaluated using mixtures of individuals of known origin, similarly as for MSA (above). We defined accuracy of the IA as the proportion of fish correctly assigned to their source population.

#### 2.4.3 Statistical analyses in R

Statistical comparisons of baseline performance were done using R (r‐project.org; R version 3.5.1). Differences between baselines in average accuracy of 100% simulation tests, self‐assignment tests, and IA‐tests with real data (TS1, TS2, TSS1 and TSS2) were investigated using Wilcoxon, Kruskal‐Wallis rank sum test and Multiple comparison test after Kruskal‐Wallis (library [pgirmess] [Giraudoux, 2013]). A 3D scatterplot with regression plane was produced using the Scatterplot3d package (Ligges & Maechler, 2003).

## 3 RESULTS

### 3.1 Genetic analysis

Among the 1,870 individuals in the original empirical baseline population samples, 96.5% had complete genotypes at all 17 microsatellites; one individual had missing data at three loci, five at two loci and 60 at one locus, resulting in overall 0.23% missing genotypes. Repeat genotyping of a subset of individuals resulted in a repeatability of 100%, and hence an estimated error rate of zero.

### 3.2 Family analysis with colony

Colony identified full‐siblings (FS) in all empirical baseline population samples consisting of two to 19 FS individuals per family. Relatively strong family structure was detected in some of the baseline population samples. For example, the sample Ljusnan (year 2006, *n* = 119) had the lowest number of families (*s* = 12) of which six consisted of 15 FS‐individuals each. We here use family to describe one “FS unit” that can include one or several individuals. The family structure of our pooled temporal baseline population samples, baseline EB1 (Large/Medium), is illustrated in Figure S1.

### 3.3 Statistical analysis

#### 3.3.1 Basic genetic analysis

No locus displayed consistent deviations from Hardy Weinberg Equilibrium (HWE) within temporal baseline population samples, and all loci were retained for further analysis. Significant deviations from HWE across loci occurred in three and two baseline population samples from 2006 and 2013/2014, respectively, in most cases as heterozygote excesses (*F*_IS_ < 0). After pooling temporal baseline population samples, significant deviations from HWE across all 17 loci (*F*_IS_ ≠ 0) was detected in one baseline population sample when all individuals were retained (baseline EB1 (Large/Medium), Table 2) and in no baseline population sample when surplus full‐siblings had been removed (baseline EB2 (Large/Weak), Table 2). There was significant genetic differentiation among baseline samples in all evaluated empirical baselines (EB1–EB4). Pairwise *F*_ST_ estimates ranged between 0.02 and 0.10, with higher estimates among baseline population samples with stronger family structure (Tables S1‐S3).

#### 3.3.2 Evaluation of baseline performance – MSA

**Figure 3:**
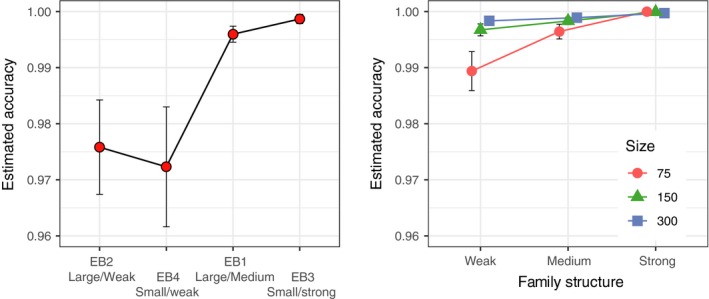
Evaluation of baseline accuracy using 100% simulations in oncor. Results from empirical (left) and simulated (right) baselines ordered by increasing family structure in baselines from left to right. Average mean accuracy across populations within each baseline (with 95% CI) is shown

The estimated mean accuracy (across populations within each baseline) of 100%‐simulations was high (>95%) and increasing with levels of family structure for all empirical and simulated baselines, although it was somewhat lower for empirical compared to simulated ones (Figure 3). The within baseline variance in estimated accuracy decreased with increasing family structure and sample size, illustrated by a decreasing (narrower) 95% CI (Figure 3).

In the empirical data, the 100% simulations showed significant differences in average estimated accuracy between baselines (Kruskal‐Wallis chi‐squared = 21.897, *df* = 3, *p* < .05) due to significant differences between EB2 (Large/Weak) & EB3 (Small/Strong), and EB3 (Small/Strong) & EB4 (Small/Weak) (multiple comparison test after Kruskal‐Wallis; *p* < .05, Figure 3). Highest average estimated accuracy was noted for the eb3 (Small/Strong) baseline (99.9%) and the lowest for the eb4 (Small/Weak) baseline (97.2%) (Figure 3).

In the simulated data, there were significant variation in average estimated accuracy determined from 100%‐simulations within each sample size class (*n* = 75, 150, 300; Kruskal‐Wallis chi‐squared, *df* = 3, *p* < .05, Figure 3), due to differences between weak and strong family structure. Significant differences in average estimated accuracy within family structure (weak, medium and strong) were also noted, indicating an effect of baseline population sample size (multiple comparison test after Kruskal‐Wallis *p* < .05, Figure 3).

**Figure 4:**
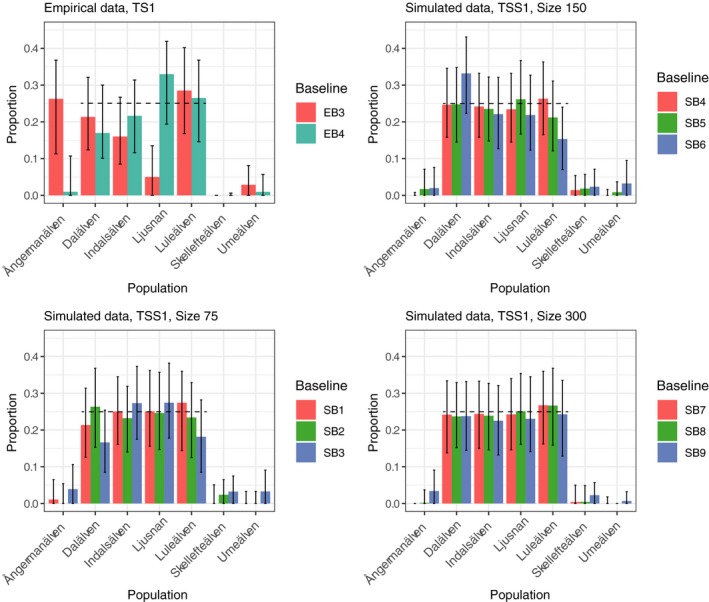
Mixed Stock Analysis (MSA) on test samples of known origin using oncor. Results from empirical (TS1, top left) and simulated (TSS1, top right and bottom) data. Black dotted lines indicate true proportions

Mixed Stock Analysis of empirical (TS1 & TS2) and simulated (TSS1 & TSS2) test mixture samples of known origin in general showed good performance, i.e., 95% CI did overlap the true stock proportions (Figure 4, Figure S6). In total, only at two out of 44 comparisons (sample Ljusnan in EB3 (Small/Strong) and sample Luleälven in SB6 (*n* = 150/Strong) the 95% CI did not overlap the true stock proportions, and in both these cases the baselines had a strong family structure. However, the MSA of TS1 & TS2 resulted in a substantial mis‐assignment of individuals to Ångermanälven when the baseline family structure was strong, i.e. EB3 (Small/Strong) (Figure 4, Figure S6).

#### 3.3.3 Evaluation of baseline performance – IA

**Figure 5:**
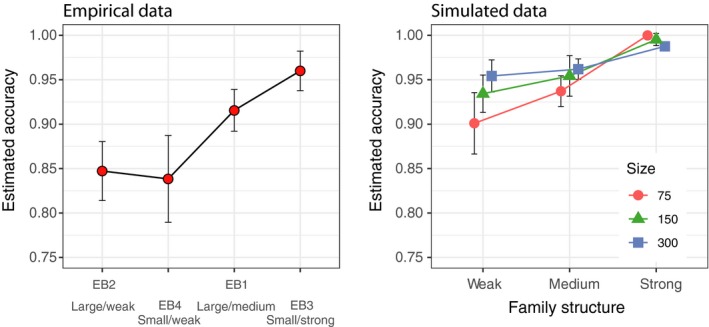
Evaluation of baseline accuracy using self‐assignment procedures (leave‐one‐out) in oncor. Results from empirical (left) and simulated (right) baselines ordered by increasing family structure in baselines from left to right. An average of accuracy across populations within each baseline with 95% CI is shown

The IA analyses showed similar results as the MSA. The self‐assignment tests for all baselines, including both empirical and simulated data, showed an increasing estimated accuracy (proportion of correctly assigned individuals) with increasing levels of family structure (Figure 5). Compared to the 100%‐simulations (see above), estimated accuracy of self‐assignment was in general lower and with wider 95% CI, and the empirical baselines showed lower estimated accuracy than simulated ones. Also, the within baseline variance in estimated accuracy decreased with increasing family structure and baseline population sample size, illustrated by a decreasing (narrower) 95% CI (Figure 5).

In the self‐assignment test of empirical data, 97.8% of individuals were assigned to a putative source of origin (oncor excluded c. 2.2% of individuals that were missing one or more genotype data, Table S8). The results from the self‐assignment tests showed significant variation in average estimated accuracy between baselines (Kruskal‐Wallis chi‐squared = 16.748, *df* = 3, *p* < .05) due to significant differences between baselines EB2 (Large/Weak) & EB3 (Small/Strong) and EB3 (Small/Strong) & EB4 (Small/Weak) (multiple comparison test after Kruskal‐Wallis, *p* < .05, Figure 5). The highest average estimated accuracy from self‐assignment was obtained for EB3 (Small/Strong) (96.0%) and lowest for EB4 (Small/Weak) (83.8%) baseline (Figure 5).

In simulated data, there were also significant variation in average estimated accuracy of self‐assignment between levels of family structure within each size class (*n* = 75, 150, 300; Kruskal‐Wallis chi‐squared, *df* = 3, *p* < .05), due to differences between weak and strong family structure. In contrast, no significant differences could be seen in estimated accuracy between baselines of different size but with the same level of family structure (multiple comparison test after Kruskal‐Wallis, *p* > .05, Figure 5).

**Figure 6:**
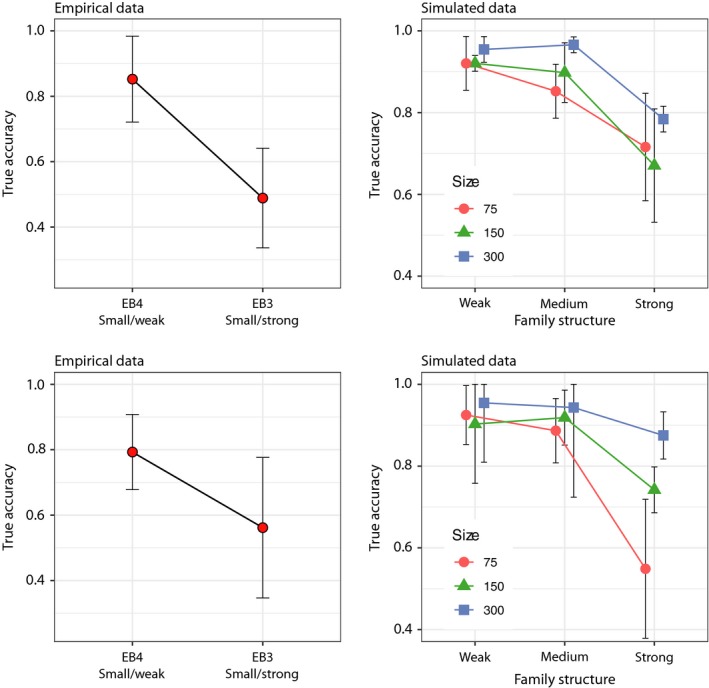
Individual assignment (IA) of two test samples of known origin using oncor. Results from empirical (TS1, top left and TS2, bottom left) and simulated (TSS1, top right and TSS2, bottom right) data, ordered by increasing family structure in baselines from left to right. An average across populations within each baseline with 95% CI is shown

The IA of mixture samples of known origin (empirical: TS1 & TS2, simulated: TSS1 & TSS2) showed results similar to the MSA of the same mixture samples, with true accuracy decreasing with increasing family structure (Figure 6). Individual Assignment for the empirical test sample TS1 showed that the weak family structure baseline EB4 (Small/Weak) resulted in a significantly higher true accuracy than the strong family structure baseline EB3 (Small/Strong) (Average true accuracy: 85% vs. 49%, Wilcoxon rank sum test; W = 15.5, *p* < .05, Figure 6). In addition, a lower number of mis‐assigned fish was noted in IA with EB4 (Small/Weak) than with EB3 (Small/Strong) (Tables S5 and S6). Similarly, the IA of TS2 resulted in on average 79% and 56% correctly assigned individuals for the eb4 (Small/Weak) and EB3 (Small/Strong) baseline, respectively (Tables S7 and S8). However this difference was nonsignificant due to large 95% CI (Wilcoxon rank sum test, *W* = 27, *p* = .18).

**Figure 7:**
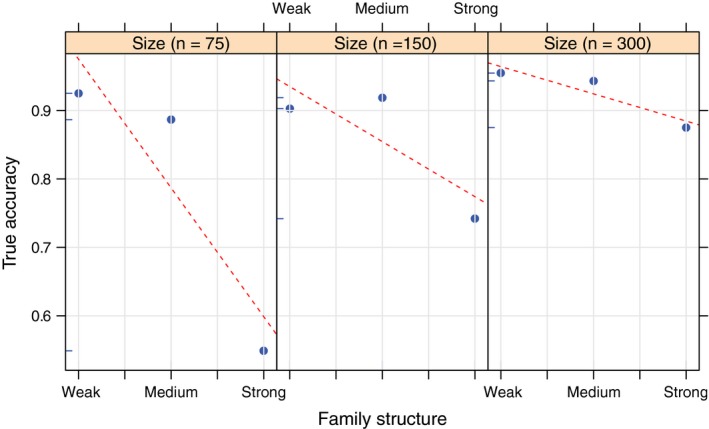
Individual assignment (IA) of test sample of known origin (TSS2) using oncor. The average accuracy against family structure by size (*n* = 75, *n* = 150, *n* = 300 for the three panels, respectively) is shown with regression line per size

**Figure 8:**
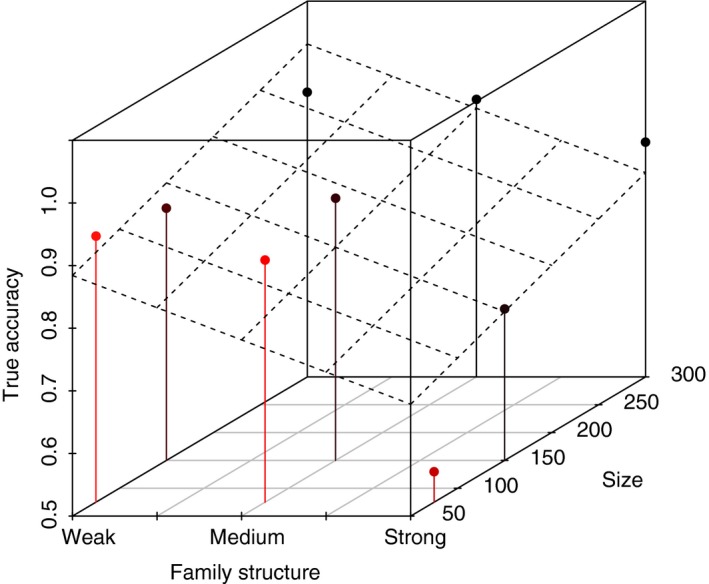
Individual assignment (IA) of test sample of known origin (TSS2) using oncor. A 3D plot with regression plane illustrating the relationship between accuracy, family structure and size. The colour is relative to sample size

Individual Assignment of simulated test samples (TSS1 & TSS2) showed a similar pattern as described above for empirical data. True accuracy decreased with increasing family structure, but in the larger baseline sample sizes (150 and 300 individuals) the decrease in true accuracy was only obvious for the strong family structure (Figure 6). Within each level of family structure, true accuracy was always highest for baselines with the largest baseline population sample size. For example, in the IA of TSS2, the average true accuracy when using a baseline with strong family structure was 55% for SB3 (Small/Strong) and 88% for SB9 (Large/Strong) (Figure 6). Plotting average accuracy versus. family structure by baseline population sample size illustrated further that the effect of increasing family structure was less pronounced at larger baseline population sample sizes (Figure 7). The relationship between true accuracy, family structure and baseline population sample size was also illustrated in a 3D plot with a regression plane (Figure 8). This showed how true accuracy decreased with increasing family structure, and at the same time that the effect was mitigated (at least partly) by an increasing baseline population sample size.

## 4 DISCUSSION

In this study, we show that nonrepresentative family sampling leading to inclusion of close relatives in a genetic reference baseline may introduce biases when evaluating the contribution of different populations in mixed samples using mixed stock analysis (MSA) and when assigning individuals to putative sources of origin using individual assignment (IA).

The influence of full‐siblings in the reference baseline was similar for analyses of both empirical and simulated genotypes, with larger bias in MSA and IA estimates with higher level of family structure. Using simulated data, we investigated if an increased baseline population sample size could mitigate this bias caused by family structure. This exercise showed that the bias was still apparent but indeed became less pronounced when baseline population sample sizes were larger. Although we did not evaluate consequences of increasing baseline population sample sizes and the number of families included, we expect that to result in even higher precision without introduced bias as we have shown that both larger sample sizes and inclusion of more families both increase true accuracy. Hence, the true accuracy of MSA and IA seems to be affected by both family structure and baseline population sample size, where a strong family structure and small baseline population sample size gives lowest true accuracy and largest bias. Interestingly, we noted that compared to IA, MSA of test samples of known origin seemed to be less sensitive to family structure since the estimated proportions with their 95% CI usually included true proportions.

The reason why family structure induced a bias in MSA and IA is most certainly related to the methodology in these assignment techniques. The MSA relate genotypes in the mixture to expected genotype frequencies in the reference baseline population samples. Similarly, IA assigns individuals in a mixture sample to the baseline population sample that would have the highest probability of producing the given genotype. If a baseline population sample consists of only a fraction of the actual population, as might be the case when family structure is present, reference allele frequencies may not be representative for the true population, resulting in biased assignment results. In particular, if baseline populations are weakly differentiated, unknown individuals might have higher likelihood to be assigned to “wrong” baseline population rather than to the biased fraction sampled from the correct one.

Another important finding in this study was that simulation tests, commonly used in GSI studies to initially evaluate accuracy and power estimates of baselines, resulted in incorrectly elevated estimates of power and accuracy when the proportion of full‐siblings in a baseline was high. For example, using the empirical baseline with strong family structure EB3 (Small/Strong) estimates of known mixtures gave a true accuracy of 20%–35% for the baseline population sample Ljusnan, whereas estimates of simulation tests using the same baseline suggested 100% estimated accuracy. Thus, accuracy was overestimated by ~70% when full‐siblings were included. The reason for high estimated accuracy in simulation and self‐assignment tests when family structure is strong is probably due to biased allele frequencies and upwardly biased differentiation among baseline population samples, which in turn will lead to higher power of such tests (e.g., Hansen et al., 2001).

We also noted from the analyses of our simulated data that 100% simulations and self‐assignment tests can be more reliable when increasing the size of the baseline population samples. In baselines with strong family structure, bias was less pronounced when using a baseline population sample size of, for example, 300 individuals compared to a baseline with only 75 individuals per baseline population sample, indicating that a larger representation of a population may improve these commonly used baseline evaluation tests. Nevertheless, our findings highlight the important contradicting results that family structure falsely improved results from accuracy tests and at the same time negatively affected GSI estimates of real data.

The question raised is how to handle full‐siblings in GSI analysis? We conclude that family structure may often induce bias, so removing excessive full‐siblings from baseline population samples appears warranted. At the same time, a large baseline population sample size is important for high accuracy of GSI estimates (e.g., Beacham et al., 2011; Hansen et al., 2001). Thus, accuracy in GSI analyses may often depend on a trade‐off between baseline population sample size and keeping family structure at a low level. Increasing representative (i.e., without surplus siblings) baseline population sample sizes seems to be the first alternative. However, researchers may often face additional complications like availability of samples, and/or costs of sampling and genetic analysis, so improving the baseline by increasing its sample sizes might not always be an option, and indeed in studies of European salmon baseline sample size is often smaller than the optimum (Beacham et al., 2011).

Waples and Anderson (2017) evaluated effects on various downstream genetic analyses (but not assignment tests) of different approaches to reduce family structure in simulated data and one empirical example. They concluded that there is no one‐size‐fits‐all method for choosing how many full‐siblings to retain. Rather they suggested that researchers should be aware of potential effects in downstream genetic analysis, which could depend on the magnitude and distribution of family structure in their samples. In addition, it was suggested that keeping two individuals per full‐sibling family seemed as a good compromise since that did not completely degrade the performance of allele frequency estimates, and yielded a higher effective sample size than when removing all but one full‐sibling per family. Waples and Anderson (2017) further made the important point that the purpose of the study in question is important, as different scientific questions might lead to different conclusions regarding handling of siblings. For example, if the purpose is to produce estimates of the effective number of breeders that produced the sampled generation, one should take a large random sample and ignore any possible family structure.

To our knowledge, this study is the first to analyze the effect of family structure on GSI estimates using empirical genetic data. We also present a complementary simulation exercise. Based on our findings we recommend that baseline population samples should always be checked for existence of close relatives so that researchers become aware of the family structure in baseline population samples and can evaluate the consequences for GSI estimates (and accuracy tests). If family structure is strong in a baseline population sample, efforts to increase the number of representative individuals is recommended. If initial sample sizes are already large (say, >150) they may be reduced by excluding excessive full‐siblings until at least a moderate family structure is achieved.

## AUTHOR CONTRIBUTIONS

J.Ö. provided the original idea and design of research; J.Ö., S.P., J.G., and J.D. further conceived and developed ideas; J.Ö. organized collection and analysis of samples; J.Ö. performed MSAs and IAs for Atlantic salmon and all statistical analysis; J.Ö. was responsible for writing the manuscript with text contributions from S.P., J.G., and J.D. All authors contributed critically to the drafts and gave their final approval for publication.

## Data Availability

Authors hereby state that upon acceptance of the manuscript for publication, data will be archived in a publicly accessible repository such as Dryad. Part of data is already deposited in the Dryad Digital Repository https://doi.org/10.5061/dryad.4pg37 (Whitlock et al., 2017).
