# Supplementary material for: Close relatives in population samples: Evaluation of the consequences for genetic stock identification
Source: Mol Ecol Resour. 2020 Jan 27;20(2):498–510. doi: 10.1111/1755-0998.13131 (PMC7065253; doi:10.1111/1755-0998.13131)
Supplement: Supplementary file 1 [file MEN-20-498-s001.docx]

# Close relatives in population samples: evaluation of the consequences for Genetic Stock Identification

Johan Östergren^1^, Stefan Palm^1^, John Gilbey^2^ and Johan Dannewitz^1^

^1^ Swedish University of Agricultural Sciences, Department of Aquatic Resources, Institute of Freshwater Research, Stångholmsvägen 2, SE-178 93 Drottningholm, Sweden

^2^ Marine Scotland Science, Freshwater Fisheries Laboratory, Faskally, Pitlochry, PH16 5LB, Scotland, U.K.

# Supporting information

Table S1. Pairwise *F*_ST_ estimates of pooled samples, i.e. empirical baseline EB1 (Large/Medium). Significant level indicated (*** = *p* < 0.001). *P*-values obtained after 21000 permutations. Indicative adjusted nominal level (5%) for multiple comparisons is: 0.002381.

|  | Ångermanälven | Dalälven | Indalsälven | Ljusnan | Luleälven | Skellefteälven | Umeälven |
| --- | --- | --- | --- | --- | --- | --- | --- |
| Ångermanälven |  | *** | *** | *** | *** | *** | *** |
| Dalälven | 0.0353 |  | *** | *** | *** | *** | *** |
| Indalsälven | 0.0218 | 0.0426 |  | *** | *** | *** | *** |
| Ljusnan | 0.0318 | 0.0270 | 0.0404 |  | *** | *** | *** |
| Luleälven | 0.0221 | 0.0310 | 0.0344 | 0.0250 |  | *** | *** |
| Skellefteälven | 0.0448 | 0.0528 | 0.0501 | 0.0549 | 0.0300 |  | *** |
| Umeälven | 0.0218 | 0.0454 | 0.0276 | 0.0358 | 0.0248 | 0.0484 |  |

Table S2. Pairwise *F*_ST_ estimates of pooled samples, i.e. empirical baseline EB2 (Large/weak). Significant level indicated (*** = *p* < 0.001). *P*-values obtained after 21000 permutations. Indicative adjusted nominal level (5%) for multiple comparisons is: 0.002381.

|  | Ångermanälven | Dalälven | Indalsälven | Ljusnan | Luleälven | Skellefteälven | Umeälven |
| --- | --- | --- | --- | --- | --- | --- | --- |
| Ångermanälven |  | *** | *** | *** | *** | *** | *** |
| Dalälven | 0.0302 |  | *** | *** | *** | *** | *** |
| Indalsälven | 0.0168 | 0.0373 |  | *** | *** | *** | *** |
| Ljusnan | 0.0236 | 0.0194 | 0.0328 |  | *** | *** | *** |
| Luleälven | 0.0194 | 0.0303 | 0.0298 | 0.0231 |  | *** | *** |
| Skellefteälven | 0.0388 | 0.0484 | 0.0438 | 0.0497 | 0.0291 |  | *** |
| Umeälven | 0.0177 | 0.0414 | 0.0216 | 0.0325 | 0.0239 | 0.0426 |  |

Table S3. Genetic differentiation (*F*_ST_) among samples in baselines EB3 (Small/Strong) and EB4 (Small/Weak). *P*-values based on 9100 permutations (indicative adjusted nominal level (5%) for multiple comparisons: 0.000549). ns = non-significant, * < 0.05, ** < 0.01). River name abbreviation as follows: Ångermanälven (AngSS, AngW), Dalälven (DalSS, DalSW), Indalsälven (IndSS, IndSW), Ljusnan (LjsSS, LjsSW), Luleälven (LulSS, LulSW), Skellefteälven (SkeSS, SkeSW), Umeälven (UmeSS, UmeSW) where SS and SW indicates samples in baselines EB3 (Small/Strong) and EB4 (Small/Weak), respectively.

|  | AngSS | AngSW | DalSS | DalSW | IndSS | IndSW | LjsSS | LjsSW | LulSS | LulSW | SkeSS | SkeSW | UmeSS | UmeSW |
| --- | --- | --- | --- | --- | --- | --- | --- | --- | --- | --- | --- | --- | --- | --- |
| AngSS |  | ns | ** | ** | ** | ** | ** | ** | ** | ** | ** | ** | ** | ** |
| AngSW | 0.0016 |  | ** | ** | ** | ** | ** | ** | ** | ** | ** | ** | ** | ** |
| DalSS | 0.0518 | 0.0448 |  | ** | ** | ** | ** | ** | ** | ** | ** | ** | ** | ** |
| DalSW | 0.0358 | 0.0295 | 0.0062 |  | ** | ** | ** | ** | ** | ** | ** | ** | ** | ** |
| IndSS | 0.0374 | 0.0298 | 0.0592 | 0.0461 |  | ** | ** | ** | ** | ** | ** | ** | ** | ** |
| IndSW | 0.0258 | 0.0180 | 0.0495 | 0.0374 | 0.0065 |  | ** | ** | ** | ** | ** | ** | ** | ** |
| LjsSS | 0.0729 | 0.0694 | 0.0682 | 0.0566 | 0.0798 | 0.0776 |  | ** | ** | ** | ** | ** | ** | ** |
| LjsSW | 0.0339 | 0.0260 | 0.0356 | 0.0248 | 0.0421 | 0.0341 | 0.0259 |  | ** | ** | ** | ** | ** | ** |
| LulSS | 0.0307 | 0.0227 | 0.0393 | 0.0285 | 0.0461 | 0.0399 | 0.0668 | 0.0305 |  | ** | ** | ** | ** | ** |
| LulSW | 0.0324 | 0.0195 | 0.0436 | 0.0312 | 0.0348 | 0.0293 | 0.0639 | 0.0278 | 0.0140 |  | ** | ** | ** | ** |
| SkeSS | 0.0723 | 0.0580 | 0.0769 | 0.0693 | 0.0775 | 0.0711 | 0.0989 | 0.0740 | 0.0540 | 0.0468 |  | ** | ** | ** |
| SkeSW | 0.0498 | 0.0382 | 0.0577 | 0.0474 | 0.0487 | 0.0407 | 0.0766 | 0.0496 | 0.0383 | 0.0292 | 0.0229 |  | ** | ** |
| UmeSS | 0.0376 | 0.0283 | 0.0590 | 0.0433 | 0.0376 | 0.0307 | 0.0696 | 0.0362 | 0.0374 | 0.0263 | 0.0679 | 0.0451 |  | * |
| UmeSW | 0.0276 | 0.0174 | 0.0586 | 0.0412 | 0.0287 | 0.0207 | 0.0709 | 0.0335 | 0.0366 | 0.0235 | 0.0633 | 0.0422 | 0.0031 |  |

Table S4. Number of individuals (*n*) in all empirical baselines (EB1-EB4) per river sample and when evaluated with Self-assignment in Oncor (only those without missing genotypes).

| Baseline EB1 (Large/Medium) |  | |
| --- | --- | --- |
|  | All individuals | Self-assignment |
| Ångermanälven | 208 | 205 |
| Dalälven | 244 | 243 |
| Indalsälven | 365 | 361 |
| Ljusnan | 333 | 301 |
| Luleälven | 275 | 270 |
| Skellefteälven | 201 | 181 |
| Umeälven | 244 | 243 |
| Baseline EB2 (Large/Weak) |  | |
|  | All individuals | Self-assignment |
| Ångermanälven | 79 | 77 |
| Dalälven | 97 | 96 |
| Indalsälven | 144 | 141 |
| Ljusnan | 135 | 132 |
| Luleälven | 131 | 131 |
| Skellefteälven | 58 | 57 |
| Umeälven | 87 | 86 |
| Baseline EB3 (Small/Strong) |  | |
|  | All individuals | Self-assignment |
| Ångermanälven | 75 | 73 |
| Dalälven | 75 | 75 |
| Indalsälven | 75 | 75 |
| Ljusnan | 75 | 70 |
| Luleälven | 75 | 72 |
| Skellefteälven | 58 | 53 |
| Umeälven | 75 | 75 |
| Baseline EB4 (Small/Weak) |  | |
|  | All individuals | Self-assignment |
| Ångermanälven | 75 | 73 |
| Dalälven | 75 | 75 |
| Indalsälven | 75 | 75 |
| Ljusnan | 75 | 75 |
| Luleälven | 75 | 75 |
| Skellefteälven | 58 | 57 |
| Umeälven | 75 | 74 |

Table S5. Results from IA for individuals of known origin (TS1) using the EB3 (Small/Strong) baseline. Correctly assigned individuals are indicated in bold.

|  |  | Donor |  |  |
| --- | --- | --- | --- | --- |
| Receiver | Dalälven (*n* = 22) | Indalsälven (*n* = 22) | Ljusnan  (*n* = 22) | Luleälven (*n* = 22) |
| Ångermanälven | 4 | 5 | 9 | 6 |
| Dalälven | **14** |  | 3 | 1 |
| Indalsälven |  | **12** | 1 | 1 |
| Ljusnan |  |  | **5** |  |
| Luleälven | 3 | 4 | 4 | **12** |
| Skellefteälven |  |  |  |  |
| Umeälven | 1 |  |  | 2 |
| Total *n* | 22 | 22 | 22 | 22 |

Table S6. Results from IA for individuals of known origin (TS1) using the EB4 (Small/Weak) baseline. Correctly assigned individuals are indicated in bold.

|  |  | Donor |  |  |
| --- | --- | --- | --- | --- |
| Reciever | Dalälven (*n* = 22) | Indalsälven (*n* = 22) | Ljusnan (*n* = 22) | Luleälven (*n* = 22) |
| Ångermanälven |  |  |  |  |
| Dalälven | **14** |  |  |  |
| Indalsälven |  | **19** |  |  |
| Ljusnan | 8 | 1 | **20** |  |
| Luleälven |  | 2 | 1 | **22** |
| Skellefteälven |  |  |  |  |
| Umeälven |  |  | 1 |  |
| Total *n* | 22 | 22 | 22 | 22 |

Table S7. Results from IA for individuals of known origin (TS2) using the EB3 (Small/Strong) baseline. Correctly assigned individuals are indicated in bold*.*

|  |  | Donor |  |  |  |  |
| --- | --- | --- | --- | --- | --- | --- |
| Reciever | Ångerman-älven (*n* = 4) | Dalälven (*n* = 8) | Indalsälven (*n* = 30) | Ljusnan (*n* = 20) | Luleälven (*n* = 14) | Umeälven (*n* = 12) |
| Ångermanälven | **4** | 2 | 6 | 6 | 4 | 4 |
| Dalälven |  | **5** |  | 3 |  |  |
| Indalsälven |  |  | **19** | 1 | 2 |  |
| Ljusnan |  |  |  | **7** |  |  |
| Luleälven |  | 1 | 5 | 2 | **6** | 1 |
| Skellefteälven |  |  |  |  |  |  |
| Umeälven |  |  |  | 1 | 2 | **7** |
| Total *n* | 4 | 8 | 30 | 20 | 14 | 12 |

Table S8. Results from IA for individuals of known origin (TS2) using the EB4 (Small/Weak) baseline. Correctly assigned individuals are indicated in bold.

|  |  | Donor |  |  |  |  |
| --- | --- | --- | --- | --- | --- | --- |
| Reciever | Ångerman-älven (*n* = 4) | Dalälven (*n* = 8) | Indalsälven (*n* = 30) | Ljusnan (*n* = 20) | Luleälven (*n* = 14) | Umeälven (*n* = 12) |
| Ångermanälven | **3** |  |  |  |  | 1 |
| Dalälven |  | **4** |  |  |  |  |
| Indalsälven |  |  | **26** | 1 | 2 |  |
| Ljusnan |  | 4 | 2 | **19** |  |  |
| Luleälven |  |  | 1 |  | **12** | 1 |
| Skellefteälven |  |  |  |  |  |  |
| Umeälven | 1 |  |  |  |  | **10** |
| Total *n* | 4 | 8 | 30 | 20 | 14 | 12 |

**Figures**


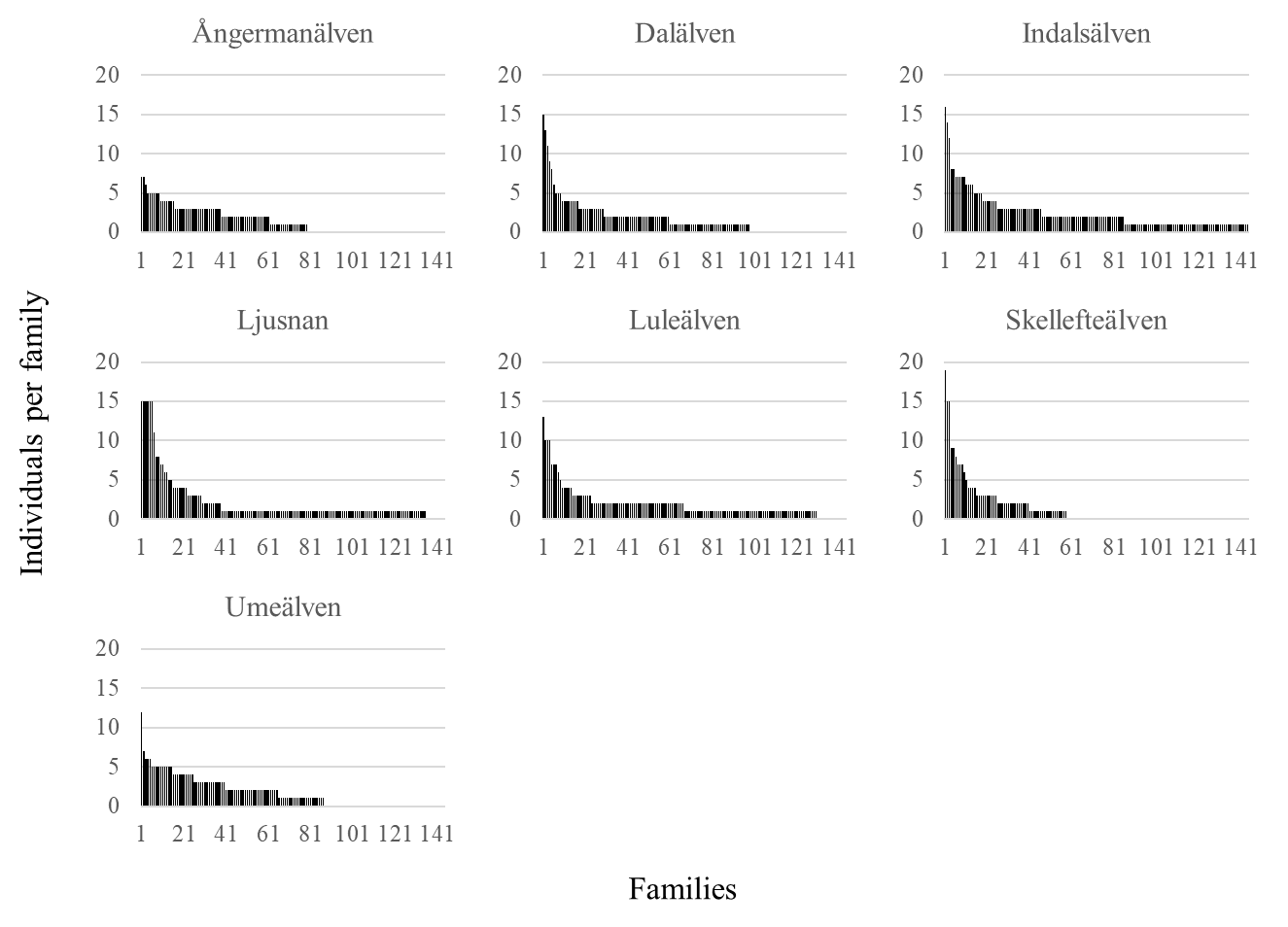


Figure S1. Number of individuals per full-sibling family in empirical baseline EB1 (Large/Medium).


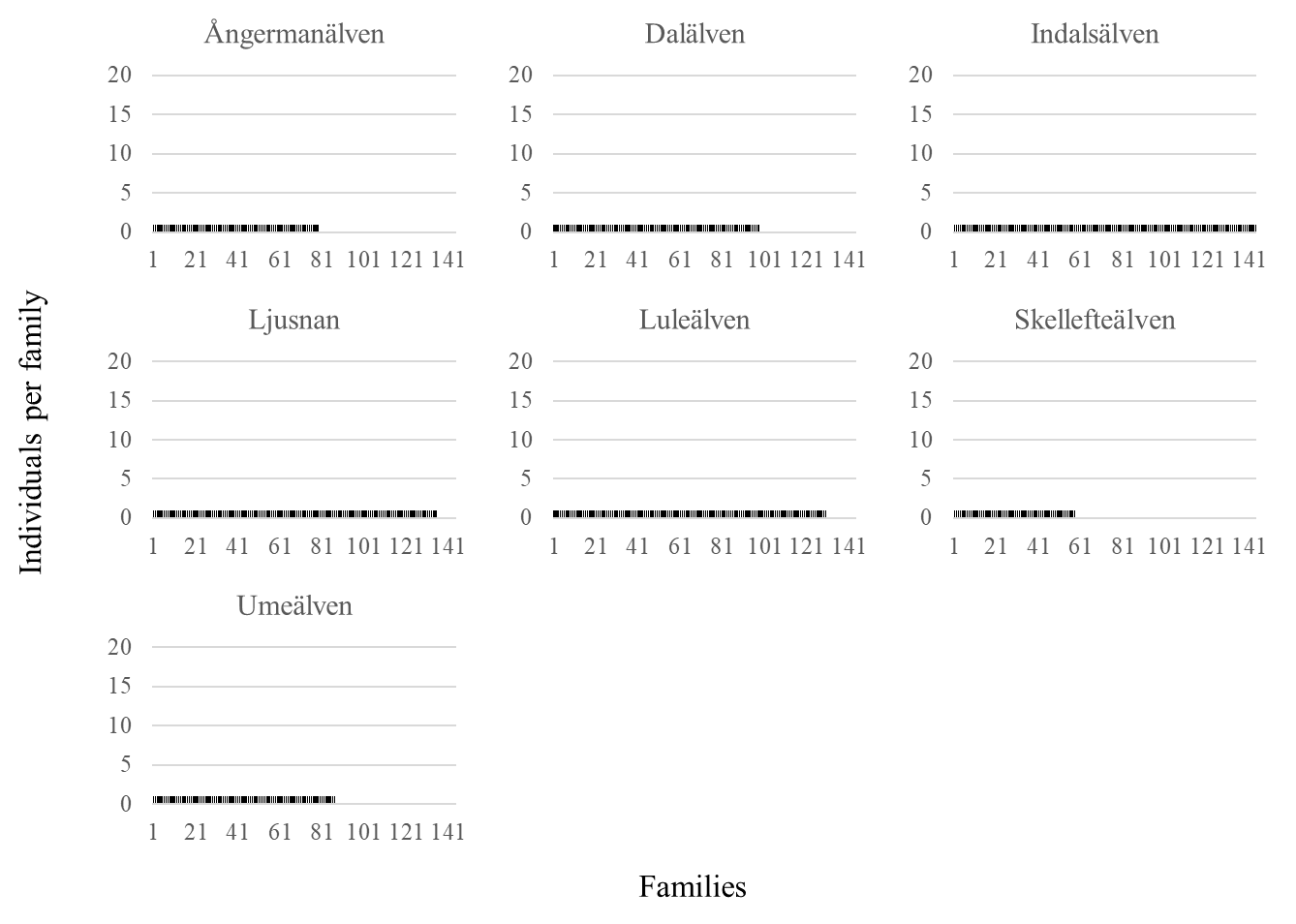

Figure S2. Number of individuals per full-sibling family in empirical baseline EB2 (Large/Weak).


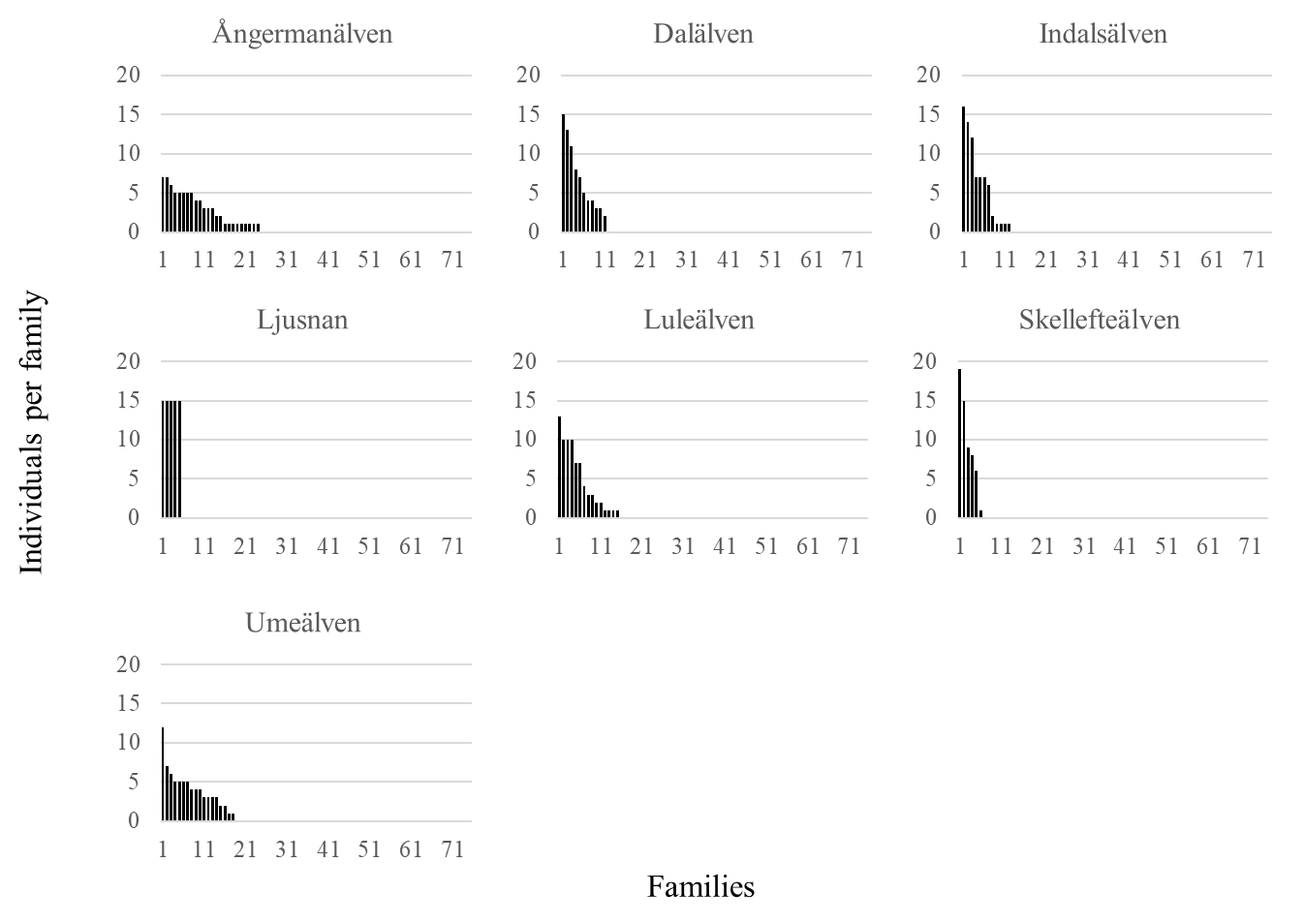


Figure S3. Number of individuals per full-sibling family in empirical baseline EB3 (Small/Strong).


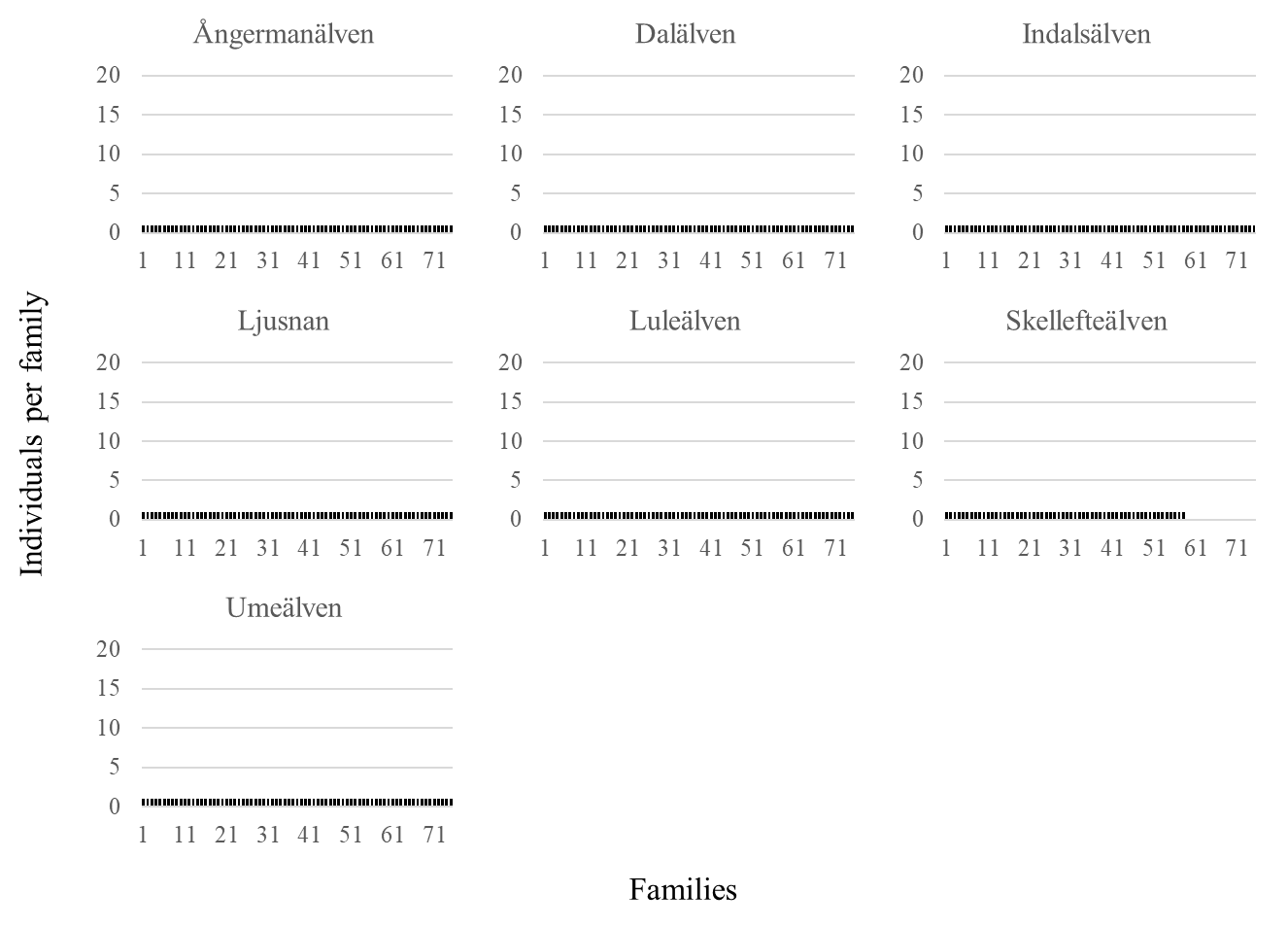


Figure S4. Number of individuals per full-sibling family in empirical baseline EB4 (Small/Weak).


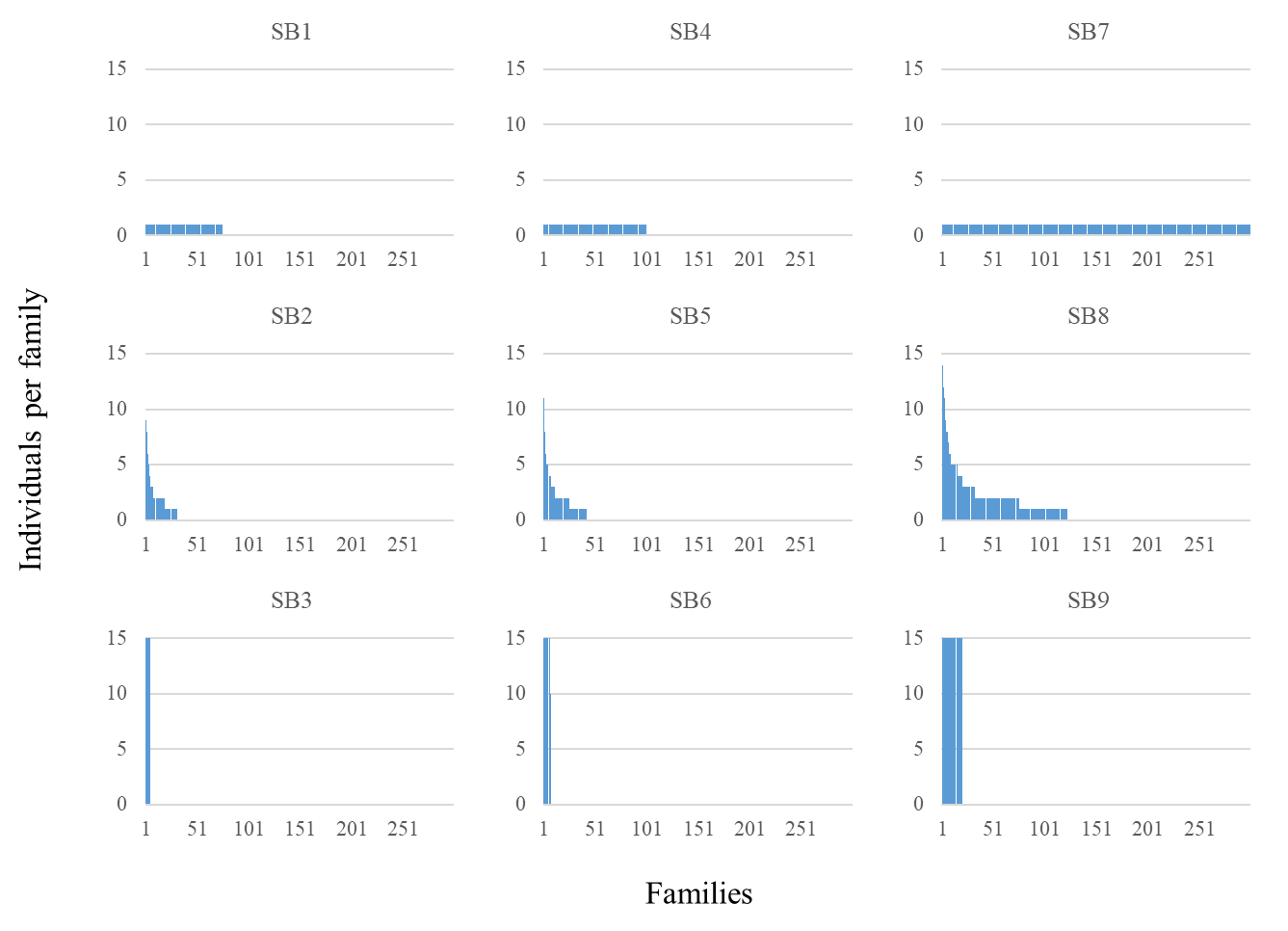


Figure S5. Number of individuals per full-sibling family in simulated baselines SB1 to SB9.


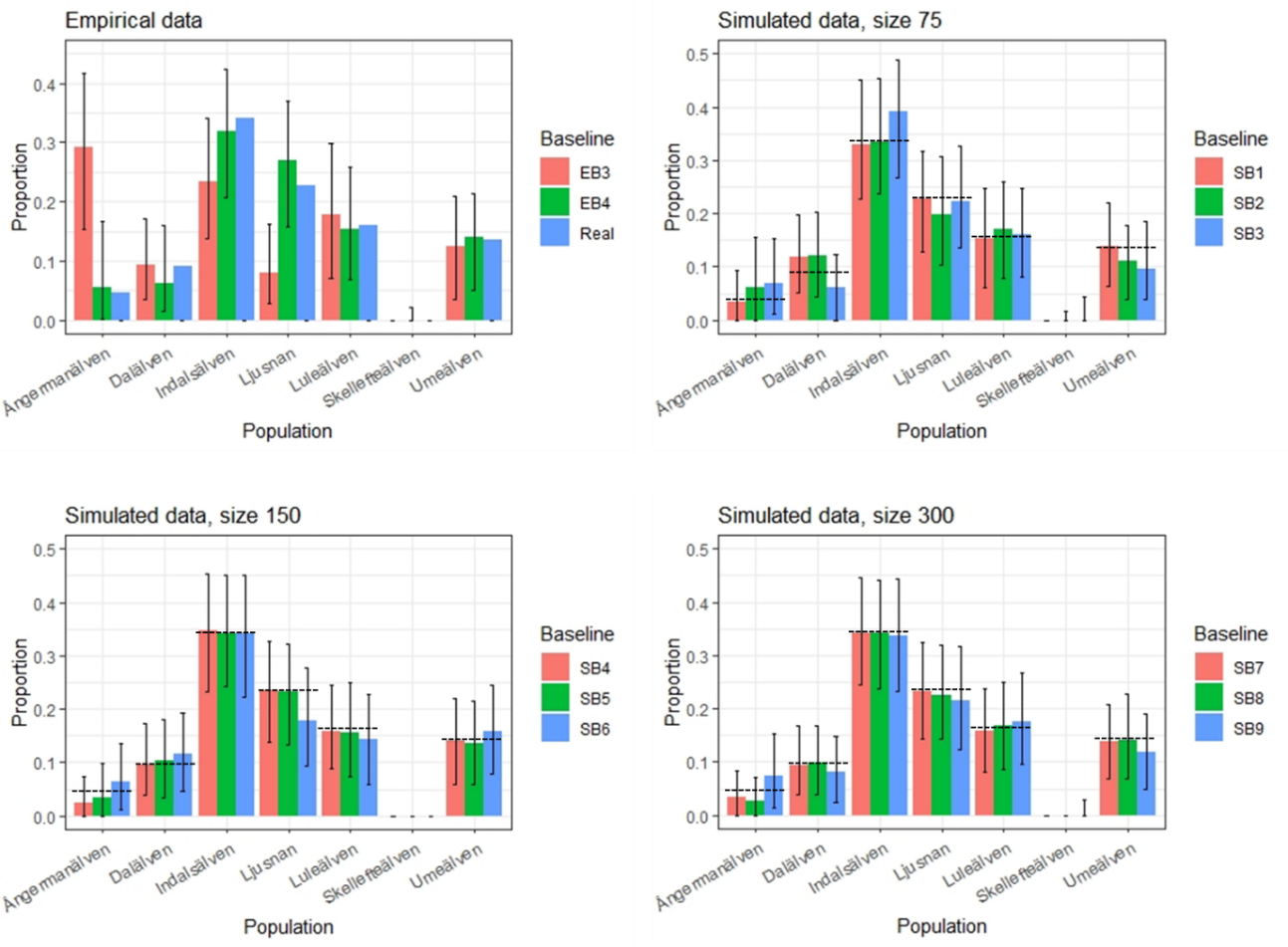


Figure S6. Results from Mixed Stock Analysis (MSA) on test samples of known origin using Oncor, divided on empirical (TS2, top left) and simulated (TSS2, top right and bottom) data. For empirical data, blue bars indicate true (real) proportions. For simulated data black dotted lines indicate true stock proportions.

# Appendix S1, Supporting information

## Statistical analysis with Colony

### Inference of family structure, i.e., full-siblings

We used the maximum likelihood approach implemented in the computer software Colony 2.0.4.4 (Wang, 2004, Wang and Santure, 2009) to identify full-siblings in each of the empirical samples (i.e. temporal samples was analyzed separately). We also used Colony to simulate baseline and test sample data for the simulation study.

Wang and Santure (2009) evaluated the performance and robustness of Colony by analyzing both simulated and empirical datasets. They showed that accuracy for sibship inferences in highly informative markers (such as microsatellites) is close to 100 % as long as a sufficient number of loci (> 12) and alleles (~100-150) are employed. Given our set of 17 polymorphic marker data, analysis with Colony should be robust when it comes to correctly identifying full-siblings (FS). Nevertheless, we tested the ability of Colony to correctly detect FS in our samples by analyzing simulated non-related offspring (*n*=100) from two hatchery populations (Ljusnan and Skellefteälven). These non-related individuals was obtain by simulating offspring using the computer software Hybridlab (Nielsen et al., 2006).

For these tests, Colony was run with different combinations of error rates (0.01, 0.001, 0.0001), likelihood precision (medium and high), length of run (medium and long), number of runs (1 or 10 replicates), and mating system (polygamy and monogamy). The result showed that, irrespective of initial settings used, Colony correctly identified 100 % and 98 % of individuals as non-related in the two unrelated simulated datasets respectively; i.e., in only a single occasion a false FS family (two full-sibs) was detected in the two simulated samples. Hence, we concluded that the risk of Colony detecting false FS families in our samples was minor, and would not significantly impact the results of our analysis. We thereafter analyzed all baseline samples using the default settings in Colony, with an error rate of 0.001 for allelic dropout, and “other” (mutation, typing error etc.). The mating system was set to mimic the procedure used in the respective hatchery and time period (mostly monogamy in both sexes). Following the analyses with Colony, we identified individuals belonging to FS families and randomly selected one individual for each FS family in each baseline sample per sampling year. This information was used to create baseline samples with and without surplus FS.

### Simulating genetic data for simulation study

To simulate the baseline samples and test samples used to evaluate GSI performance we used allele frequencies from the baseline E2 (Large/Weak). Colony was set with default initial parameters, different family matrixes to obtain the different family structures used in the simulation study (see below), a zero error rate, and empirical allele frequencies for the samples in the baseline or test samples, respectively. In total, 63 baseline samples was simulated (nine baselines of seven samples), and two test samples.

Baseline samples consisted of three levels of family structure (weak, medium and strong) and three levels of sample size (75, 150 and 300 individuals per baseline sample). The family structure was defined as follows; weak – the same as in the empirical study (i.e. non-related individuals), medium – was based on the average family structure in baseline E1 (Large/Meduim), and strong – was based on the strongest family structure in the empirical data, which was an equal family size of 15 individuals per baseline sample. The family structure in S1–S9 is illustrated in figure S5, Supporting information.

The two test samples (TSS1–TSS2) consisted of non-related individuals simulated using allele frequencies in the baseline E2 (Large/Weak). Similar to the empirical study, TSS1 consisted of equal number of individuals (*n* = 22) from four populations and TSS2 consisted of unequal number of individuals (*n* = 4–30) from six populations.

NIELSEN, E. E., BACH, L. A. & KOTLICKI, P. 2006. HYBRIDLAB (version 1.0): a program for generating simulated hybrids from population samples. *Molecular Ecology Notes,* 6**,** 971-973.

WANG, J. & SANTURE, A. W. 2009. Parentage and Sibship Inference From Multilocus Genotype Data Under Polygamy. *Genetics,* 181**,** 1579-1594.

WANG, J. L. 2004. Sibship reconstruction from genetic data with typing errors. *Genetics,* 166**,** 1963-1979.
